# Childhood emotional problems and self-perceptions predict weight gain in a longitudinal regression model

**DOI:** 10.1186/1741-7015-7-46

**Published:** 2009-09-11

**Authors:** Andrew Ternouth, David Collier, Barbara Maughan

**Affiliations:** 1 Social, Genetic and Developmental Psychiatry Centre, Institute of Psychiatry, King's College London, UK

## Abstract

**Background:**

Obesity and weight gain are correlated with psychological ill health. We predicted that childhood emotional problems and self-perceptions predict weight gain into adulthood.

**Methods:**

Data on around 6,500 individuals was taken from the 1970 Birth Cohort Study. This sample was a representative sample of individuals born in the UK in one week in 1970. Body mass index was measured by a trained nurse at the age of 10 years, and self-reported at age 30 years. Childhood emotional problems were indexed using the Rutter B scale and self-report. Self-esteem was measured using the LAWSEQ questionnaire, whilst the CARALOC scale was used to measure locus of control.

**Results:**

Controlling for childhood body mass index, parental body mass index, and social class, childhood emotional problems as measured by the Rutter scale predicted weight gain in women only (least squares regression *N *= 3,359; coefficient 0.004; *P *= 0.032). Using the same methods, childhood self-esteem predicted weight gain in both men and women (*N *= 6,526; coefficient 0.023; *P *< 0.001), although the effect was stronger in women. An external locus of control predicted weight gain in both men and women (*N *= 6,522; coefficient 0.022; *P *< 0.001).

**Conclusion:**

Emotional problems, low self-esteem and an external locus of control in childhood predict weight gain into adulthood. This has important clinical implications as it highlights a direction for early intervention strategies that may contribute to efforts to combat the current obesity epidemic.

## Background

Associations between body mass index (BMI) and a range of psychiatric/psychological vulnerabilities are now well established. Cross-sectional studies at different stages in the life course have shown, for example, that obesity and depression co-occur more frequently than expected by chance [1-3], and there are similar findings for anxiety [4]. Individuals with high BMI [5,6], or with bulimia or binge-eating are likely to have low self-esteem [7-10], and to be at particular risk of developing psychological comorbidity along with obesity [11]. The relationships between obesity, BMI, and psychological morbidity are also thought to differ between sexes [1,12,13]. Depressive symptoms are commonly found to be associated with overweight or obesity in women, whilst results in men suggest an inverse or non-significant relationship.

While this broad pattern of associations is now generally accepted, debate continues over the direction of the influences involved. In the short term, associations are likely to be complex: weight problems have been found to influence mood states and self-perceptions in some studies [14], but not others [15], whilst depression has also been shown to predict weight gain, and both mediating and moderating influences have also been proposed [16]. In principle, prospective studies over more extended developmental periods should help to clarify the direction of effects involved, and long-term longitudinal evidence of this kind has now begun to be reported. Childhood temperament, for example, has been shown to predict adult BMI [17], with negative emotionality at ages 6 to 10 years predicting an increased BMI in early adulthood. Indeed, negative emotionality has been associated with an increase of weight gain even in infancy [18], and has also been identified as a risk factor for eating disorders in early teenage girls [19]. Other evidence suggests that depression during childhood or adolescence, but not adulthood, is positively associated with later obesity [20,21], especially amongst women [22].

However, not all past findings have been consistent with this view. In particular, Viner and Cole [23], using data from the British 1970 Birth Cohort Study (BCS70), found that whilst age 16 activity levels and eating behaviour significantly predicted BMI at age 30, there was no significant effect of psychological measures (depressed mood and self-esteem) assessed in adolescence. As many adolescents experience periods of moodiness and low self-esteem, which relate to developmental changes, we speculated that any associations with later outcomes might be less evident in the teens than at earlier developmental periods. To test this, we returned to the BCS70 , but focused on measures collected at the 10-year assessments to investigate whether childhood emotional problems (teacher-reported emotional difficulties and self-reported nervousness) or self-perceptions (specifically self-esteem) predict weight gained in adulthood. BCS70 has a number of advantages for a study of this kind. In particular, its prospective design allows clear tests of temporal ordering, and the wealth of other measures available allows controls for likely confounds. Childhood obesity, for example, often continues into adulthood [24], and low childhood socio-economic status (SES) [25] and parental obesity [26] are well known to predict higher adult BMI. As measures of these constructs were also available in childhood sweeps of BCS70, we were able to assess links between childhood emotional difficulties and weight gain net of the effects of these and other potential confounds.

In addition to emotional problems and self-esteem, the age 10 assessments in BCS70 also included measures of one further construct of interest in relation to eating behaviours, that is, locus of control. Locus of control concerns individuals' perceptions of the extent to which their own actions affect their destiny. Those with an internal locus of control believe that their behaviours have an important influence on outcomes, whereas those with a more external locus of control believe that their fate lies more in the hands of others, or is dependent on circumstance. Several reports have now associated weight status or eating disorders with a more external locus of control. In a cross-sectional study, obese women were found to have a more external locus of control than women with normal weight [27]. People with a more internal locus of control have been shown to be more physically active than others [28], and a more external locus of control has also been linked with bulimia nervosa [29-31], anorexia nervosa [32], as well as disordered eating behaviours [33] such as binge eating [34]. An external locus of control could be related to low self-efficacy, a lack of belief in one's own capabilities, which in turn could lead to a feeling of loss of control of one's actions. A loss of control of eating behaviour can contribute to overweight in childhood [35], and it is possible that this behaviour will continue, resulting in overweight later in the life course. Self-efficacy in avoiding weight gain has also been shown to predict BMI at 2-year follow-up in young women [36]. However, it has also been suggested that locus of control may have a mediating effect on weight, and one study suggests that an external rather than internal locus of control has a more positive health outcome [37]. Recently the relationship between childhood locus of control and adult BMI was examined in the BCS70 cohort [38], and an association was shown between higher adult BMI and a childhood external locus. So far as we are aware, no studies have yet examined associations between locus of control in childhood and weight gained by age 30 years.

Against this background, our study had two main aims. First, we hypothesised that children with greater emotional difficulties at age 10 years would experience an increased weight gain into adulthood when compared with their peers. Second, we hypothesised that children who had lower self-esteem and a more external locus of control would have a larger gain in weight tracking into adulthood, when compared with their peers. In light of past findings we also anticipated that these associations would be stronger in women than in men.

## Methods

### Sample

Data were taken from the BCS70 [39]. This prospective study includes 16,496 children born in England and Wales in one week in April 1970. Cohort members have been studied at a range of ages across childhood and into adulthood: here we utilise data taken at birth and at ages 10 and 30 years. High response rates were achieved at both the age 10 (89.7%) and age 30 (70.1%) follow-ups [40]. We included 7,588 individuals (51.1% female) in the current study. Individuals were excluded from the analysis if they did not have BMI information at age 30 (5,566 individuals), BMI at age 10 (863 individuals), paternal BMI information (2,168 individuals), maternal BMI information (111 individuals), social class information at age 10 (196 individuals), or ethnicity information at age 10 (4 individuals). Tests for effects of attrition showed that exclusion from the analysis was predicted by male sex, and that there were slight variations in BMI at age 10 (equivalent to 0.012 standard deviation units), study members with lower BMI scores being slightly less likely to be included. Inclusion in the final sample was not associated with parental BMI, social class or ethnicity. Non-response weights were derived from the inverse of the predicted values from a logistic regression analysis predicting attrition, and were used in the analyses throughout.

### Measures

#### Demographic background

Childhood social class was determined from reported parental occupation data at the age 10 contact and coded on a six-point scale according to the Registrar General's classification system.

Ethnicity was determined from parental report at child age 10. Parents were asked to indicate to which category the child belonged: (a) English, Welsh, Scottish and Northern Irish; (b) Irish; (c) other European; (d) West Indian; (e) Indian; (f) Pakistani; (g) Bangladeshi; (f) other. As the vast majority of subjects (97.4%) belonged to groups (a) to (c), categories were collapsed and ethnicity was coded as 'European' or 'Non-European' during the analysis.

#### BMI

Age 10: participants' height (mm) and weight (g) were measured by a trained nurse at age 10. These units were converted to kilograms and metres and BMI was calculated as weight/height^2^. This measure was then standardised to produce a zBMI (standardised BMI) score.

Age 30: at age 30, individuals self-reported their current height and weight. All units were converted to metric measures and BMI was again calculated as weight/height^2^.

Parental BMI: the height and weight of the study members' father and mother were reported by the main parental respondent at the age 10 contact. These measures were converted to metric units, and BMI was calculated as weight/height^2^, which was then standardised.

#### Childhood emotional problems

Teacher reports: teachers completed a slightly modified version of the Rutter B(2) scales [41] at the age 10 study contact. We used four items (worried, miserable, fearful, fussy) to index emotional difficulties (Cronbach alpha = 0.81). Each item was marked on a visual scale to generate a continuous score ranging from 1 to 47 with a mean score used in the analysis. Emotional problem sub-scales of the Rutter scales show good predictive validity; in the current sample, children scoring in the top 10% of the range on the emotional problems scale had an around 60% increased risk of consulting a specialist for emotional difficulties/depression in adolescence and early adulthood (odds ratio 1.63, 95% confidence intervals 1.39-1.92).

Self-reports: children completed self-report questionnaires at the age 10 study contact including two items indexing worrying/nervousness ('I worry a lot', 'I am nervous'), Possible responses were 'sometimes', 'often or usually' or 'not at all' (scored 1, 2 and 3).

#### Childhood self-perceptions

Self-esteem: the children also completed the LAWSEQ measure of self-esteem [42] at age 10. This validated measure [43] comprises 12 items, each item answered 'yes', 'don't know' or 'no'. 'Yes' answers were coded as 1, with 'no' and 'don't know', coded as 0 and item responses summed to give a total score from 0 (high self-esteem) to 12 (low self-esteem, Cronbach's alpha = 0.66). EM imputation procedures were used (using the EM data imputation in SPSS) to generate total scores in cases with at least 8 but fewer than 12 valid responses (5.4% of the sample).

Locus of control: at age 10 the locus of control was assessed using the CAROLOC scale developed for use in the BCS70 [44]. This scale measures whether an individual's locus of control is external or internal (across a continuum). Sixteen items such as 'do you feel that most of the time it's not worth trying hard because things never turn out right anyway?', 'are you the kind of person who believes that planning ahead makes things turn out better?' and 'when bad things happen to you, is it usually someone else's fault?' were presented. Possible answers were 'yes' (coded 1), 'no' and 'don't know' (coded 0). Item responses were summed to give a total score from 0 (internal) to 16 (external), (Cronbach's alpha = 0.62). EM imputation procedures (using the EM data imputation in SPSS) were used to generate total scores in cases with at least 12 but fewer than 16 valid responses (5.9% of the sample). As Gale *et al. *[38] outlined, the Caraloc locus of control scale was initially devised to assess locus of control in relation to school performance - a key issue in middle childhood. Past studies have reported associations between contemporaneously assessed locus of control and health-related behaviours in adult samples. Consistent with these expectations, Gale *et al. *[38] found that Caraloc scores remained significant predictors of all the adult self-reported health outcomes they examined, after full adjustment for educational attainment, IQ, social class and earnings, suggesting that this scale acts as a valid measure of the sense of inner control in childhood.

### Statistical methods

All statistical analysis was carried out using STATA. Tests for mean differences in continuous measures were conducted using standard *t*-tests, and tests for differences in categorical variables using logistic regression or ordinal logistic regression as appropriate. Pair-wise correlations were used to investigate relationships between confounding variables and the independent variable measures of self-perception and emotionality. Ordinary least squares regression was used to examine predictors of the main continuous outcome variable (BMI at age 30).

## Results

### Sample characteristics

Table 1 shows descriptive data on sample characteristics in childhood for the full sample, and for men and women separately. As expected there were no sex differences in childhood social class or ethnicity or in parental zBMI. zBMI at age 10 was significantly higher amongst girls, but by age 30 this sex difference was reversed. Teachers rated 10-year-old girls as showing significantly higher levels of emotional difficulties than boys, and self-reports at age 10 also identified higher levels of nervousness and worrying among girls. Boys had a more external locus of control and higher self-esteem than girls at age 10. Of the 7588 individuals selected for analysis, data on measures of emotionality and self-perceptions were available for 6384-6567 individuals.

**Table 1:** Sample characteristics

			***Mean/Proportion***			***Sex differences***	
	**Variable**	**Group**	**Total Sample** **(n = 7588)^1^**	**Men** **(n = 3709)^1^**	**Women** **(n = 3879)^1^**	**t-test p value**	**Rank Sum P value^2^**
*Demographic Variables*	Social Class						0.690
		% non-manual	31.67	31.46	31.88		
		% manual	68.33	68.54	68.12		
	Ethnicity						0.371
		% European	97.37	97.20	97.53		
		% Non - European	2.63	2.80	2.47		
*Study Member BMI*	age 10		0.01	-0.07	0.09	*0.000	
	age 30		-0.01	0.14	-0.15	*0.000	
*Parental B M I*	Father		-0.03	-0.03	-0.02	0.600	
	Mother		-0.03	-0.04	-0.02	0.377	
*Childhood Emotional Problems*	Rutter Emotional score Self Reported Nervousness		14.41	13.66	15.11	*0.000	*0.000
		% Not at all	12.07	15.05	9.27		
		% Sometimes	82.02	79.60	84.29		
		% Often	5.91	5.35	6.44		
	Self Reported Worrying						*0.000
		% Not at all	27.09	30.53	23.86		
		% Sometimes	60.25	57.91	62.45		
		% Often	12.66	11.57	13.69		
*Psychological Measures*	CAROLOC Locus of control		4.40	4.53	4.28	*0.000	
	LAWSEQ self esteem		3.41	3.18	3.64	*0.000	

^1^Numbers vary slightly as a result of occasional missing data, sample sizes are the same as those listed in table 3.

^2^Rank Sum P value refers to a Two-sample Wilcoxon rank-sum (Mann-Whitney) test. * significant p value (p < 0.05)

### Identifying potential confounders

We used simple linear regression models to test bivariate associations between BMI at age 30 and a range of background characteristics. As expected, adult BMI showed strong continuities with zBMI in childhood (beta = 0.39, *P *< 0.001). BMI at age 30 did not vary significantly between ethnic groups (beta = -.036, *P *= 0.608), but was significantly predicted by sex (beta = 0.29, *P *< 0.001), childhood social class (beta = 0.06, *P *< 0.001), paternal zBMI (beta = 0.19, *P *< 0.001) and maternal zBMI (beta = 0.22, *P *< 0.001). These factors were treated as confounders in subsequent analyses with regression models including these terms along with each independent variable. The correlation between these confounders and measures of emotionality and self-perception are seen in Table 2. Social class was significantly correlated with subject and parental BMI, as well as self-perception and Rutter-scored emotionality. Subject BMI at age 10 was correlated with parental BMI as well as emotional problems, but was not significantly correlated with self-perception measures. Parental BMI itself appeared to be significantly correlated with the child's locus of control and perhaps self-esteem (maternal BMI only), but was not significantly correlated with scores of emotional problems. The measures of emotionality and self-perception were all significantly correlated with one another.

**Table 2:** Correlations between confounding variables and measures of emotionality and self perception

	**Social Class**	**BMI age 10**	**Paternal BMI**	**Maternal B MI**	**Rutter emotional score**	**Self Reported Nervousness**	**Self Reported Worrying**	**CAROLOC Locus of Control**	**LAW SEQ Self Esteem**
Social Class	1								
BMI age 10	*0.026	1							
Paternal B MI	*0.110	*0.196	1						
Maternal BMI	*0.144	*0.215	*0.163	1					
Rutter emotional Score	*0.066	*-0.025	0.002	0.019	1				
Self Reported Nervousness	0.016	*0.030	0.011	0.018	*-0.070	1			
Self Reported Worrying	-0.006	*0.026	-0.003	-0.019	*-0.103	*0.278	1		
CARO LOC Locus of Control	*0.186	0.015	*0.064	*0.083	*0.162	*-0.054	*-0.115	1	
LAW S EQ Self Esteem	*0.095	0.010	0.017	*0.060	*0.179	*-0.210	*-0.276	*0.424	1

*Significant *P *value (*P *< 0.05).

### Weight gain and childhood emotional problems

Results of multiple regressions assessing the extent to which childhood emotional difficulties and self-perceptions are predictive of BMI at age 30 taking account of sex, childhood BMI, parental BMI and social class (including childhood BMI allows us to estimate weight gain) are displayed in Table 3. The left-hand panel shows results for the whole sample, whilst the other panels show results for males and females separately, along with results of tests for interactions by sex. Table 3 presents *P *values uncorrected for multiple testing. Whilst multiple testing is an important concern, here we perform a number of non-independent tests and subsequently do not apply an overly conservative Bonferonni correction. In practice, however, many of the *P *values presented in Table 3 would survive such correction.

**Table 3:** Childhood emotional problems, childhood self perceptions, and adult BMI

	**Category**	**Males and Females**				**Males**				**Females**				**Sex interaction**	
**Independent Variable**		**N**	**Coefficient**	**r2**	**P Value**	**N**	**Cofficient**	**r2**	**P Value**	**N**	**Coefficient**	**r2**	**P Value**	**Coefficient**	**P value**
Rutter Emotional Score		6567	0.007	0.216	0.250	3208	-0.007	0.171	0.446	3359	0.019	0.224	*0.032	-0.028	*0.024
Self Reported Nervousness		6482		0.217		3157		0.172		3325		0.224			
	Often	383	0.373		0.079	169	0.304		0.294	214	0.484		0.108		
	Not at all	783	0.336		*0.040	475	0.304		0.088	308	0.326		0.219		
Self Reported Worrying		6384		0.217		3112		0.171		3272		0.225			
	Often	808	0.358		*0.020	360	0.246		0.268	448	0.447		*0.035	-0.263	0.391
	Not at all	1730	0.217		0.078	950	0.289		0.074	780	0.103		0.583	0.181	0.465
CAROLOC Locus Of Control		6528	0.107	0.220	*0.000	3190	0.069	0.174	*0.011	3338	0.147	0.229	*0.000	-0.109	*0.007
LAWSEQ Self Esteem		6552	0.102	0.219	*0.000	3203	0.069	0.173	*0.027	3349	0.128	0.228	*0.000	-0.017	0.086

*significant p value (p < 0.05)

Results are controlled for sex, social class, z BMI at age 10, and parental BMI, r^2 ^values denote variance accounted for by the regression model including the independent variable and these confounders (5 models total).

Controlling for sex, childhood BMI, parental BMI and social class, teacher reported emotionality was unrelated to BMI at age 30 in the sample as a whole, and amongst men, but increased emotionality was significantly associated with higher adult BMI amongst women; the test for an interaction by sex was statistically significant. Analyses of the three-category measures of self-reported nervousness and worrying contrasted study members who reported no problems on the one hand, or frequent problems on the other, with the majority of the sample who responded that they 'sometimes' experienced difficulties of these kinds, whilst controlling for sex, childhood BMI, parental BMI and social class. As Table 3 shows, frequent self-reported nervousness at age 10 did not significantly predict adult BMI in either male- or female-only samples, although there was a non-significant tendency towards higher BMI in this group across the sample as a whole. Ten-year-olds who reported not being at all nervous in childhood, however, were predicted a significantly higher BMI in the adult sweep, although again this effect failed to reach conventional significance levels when the sexes were analysed separately. In relation to worrying, study members who reported that they did not worry at all in childhood were not predicted a significantly different adult BMI than those who sometimes worried, but in both the whole sample, and in the female-only sample, those who reported often worrying were predicted a higher BMI aged 30. There was no significant sex interaction.

### Weight gain and childhood self-perceptions

Table 3 shows parallel results for the measures of locus of control and self-esteem in childhood. In male-only, female-only, and the whole sample, a more external locus of control in childhood was shown to predict significantly a higher BMI in adulthood, whilst controlling for sex, childhood BMI, parental BMI and social class. There was also a significant interaction between sex and locus of control, with prediction being stronger for women than for men. Controlling for sex, childhood BMI, parental BMI and social class, lower childhood self-esteem significantly predicted a higher adult BMI in the whole sample, and in both male- and female-only samples. Tests for an interaction between sex and self-esteem fell short of conventional significance levels, although there was a suggestion that the effect might be larger in girls.

Thus far, we have shown that a number of indicators of childhood emotional problems/self-perceptions are related to weight gain, with some of these associations being stronger for women than for men. To identify which indicators showed independent effects we undertook two final analyses (one for women and one for men), in which all significant predictors and confounders were included in a single model (Tables 4 and 5). We used standardised measures in order to indicate the relative importance of confounders and predictors. The full male regression model contained confounders, the CAROLOC locus of control and LAWSEQ self-esteem. When both of these measures were included in the model neither was significant, although locus of control approached significance (*P *= 0.070). This model accounted for 17.5% of the variance in BMI at age 30. The full female regression model contained confounders, the Rutter scale of emotionality, self-reported worrying, CAROLOC locus of control and LAWSEQ self-esteem. Of these measures, both the locus of control and self-esteem remained independent significant predictors of weight gain. This model accounted for 23.0% of the variance in BMI at age 30.

**Table 4:** Coefficients and p values for the full male standardized multiple regression model

**Variable**	**N**	**zCoefficient**	**P Value**
	3186		

Social Class		0.002	0.897

Paternal BMI		0.079	0.000

Maternal BMI		0.065	0.000

BMIage 10		0.358	0.000

CAROLOC Locus of Control		0.029	0.071

LAWSEQ Self Esteem		0.023	0.171

Results are controlled for social class, zBMI at age 10 and parental BMI. The model uses standardised variables in one multiple regression model.

**Table 5:** Coefficients and *P *values for the full female standardised multiple regression model

**Variable**	**Category**	**N**	**zCoefficient**	**P Value**
		3230		

Social Class			0.041	0.030

Paternal BMI			0.110	0.000

Maternal BMI			0.167	0.000

BMI age 10			0.347	0.000

Rutter Emotional Score			0.022	0.186

Self Reported Worrying				

	Often		0.052	0.274

	Not at all		0.051	0.224

CAROLOC Locus Of Control			0.059	0.009

LAWSEQ Self Esteem			0.039	0.044

Results are controlled for social class, zBMI at age 10 and parental BMI. The model uses standardised variables in one multiple regression model. Results are controlled for social class, zBMI at age 10 and parental BMI. The model uses standardised variables in one multiple regression model.

## Discussion

We hypothesised that children with emotional difficulties, low self-esteem, and an external locus of control would exhibit a larger weight gain tracking into adulthood when compared with their peers. Even when controlling for childhood SES and parental BMI, these hypotheses were supported by the data. In the whole cohort, self-esteem, self-reported worrying, self-reported nervousness and locus of control all significantly predicted weight gain. In the female group only, Rutter scale emotionality significantly predicted weight gain. There was a significant interaction between emotionality and sex, and also between locus of control and sex, with the effect size in each case being greater in females than in males. However, when analysing these early predictors jointly in sex-specific models, only locus of control and self-esteem remained as significant predictors of weight gain, and then only in females. As expected, the strongest predictors of weight gain were BMI at age 10 and parental BMI. However, locus of control and self-esteem at age 10 predicted weight gain on par with social class.

The representative nature of the BCS70 cohort makes it especially suitable for testing the hypotheses proposed in the introduction. Beyond this, it has a number of other advantages and also some potential limitations. That it is drawn from all people born in the UK in a particular week in 1970 is advantageous, in that the members of the cohort should have been be exposed to similar sociocultural trends over their lifespan; by the same token, it is inevitably limited in its capacity to assess the effect of sociocultural changes occurring between cohorts born in different years. Season of birth could also have had a small effect on the pattern of the findings, as all BCS70 cohort members were born in the spring. However, the sample was large, provided a long-term period of follow-up, and included measures of BMI in both childhood and adult life. The measures of BMI in childhood are particularly accurate as they were measured by a trained practitioner. We recognise that there may be some inaccuracies in self-reported body weight at the age 30 sweep [45], although the adults in this cohort were younger and may consequently have fewer self-reporting errors than have been found in studies of older adults [46]. Indeed a report bias of participants under-reporting BMI at higher BMI would make the results estimated conservative. As with all longitudinal studies, the numbers of participants were smaller in later sweeps, with males somewhat underrepresented in the analysed samples. Age 10 BMI was also significantly, but very slightly associated with availability of complete data. We used weights to correct so far as possible for any biases associated with attrition. The authors are unaware of any further biases which may have affected the results.

Although the psychological measures used by Viner and Cole at age 16 [23] did not predict adult BMI in this sample, here we showed that similar measures taken at age 10 are predictive. Two factors may have contributed to these variations. First, from a developmental perspective, the 'storm and stress' of adolescence may mean that mood states and self-cognitions in the mid-teens are less stable markers of individual differences in weight-related psychological features than comparable measures taken in late childhood. Second, like Viner and Cole [23], we found that measures of emotional difficulties did not show independent links with change in BMI, but that self-perceptions and self-cognitions did. As outlined above, there is already a body of literature associating self-esteem with BMI in cross-sectional studies. Although bidirectional influences are likely to be involved here, our findings, based on prospective data spanning an extended follow-up period, do lend support to the possibility that poor childhood self-esteem contributes to later risk for high adult BMI. Consistent with the locus of control literature, our findings also suggest that an external locus of control may contribute to the development of poor dietary, exercise or eating habits, in the belief that these actions will not affect outcome. It is possible that there is simply less effort to control weight due to a fatalistic or deterministic world view. Recently Gale *et al. *[38] showed that an external locus of control in childhood predicts absolute weight in adulthood; consistent with the idea that an external locus of control leads to poorer dietary habits, our findings suggest that an external locus of control also predicts weight gain.

From this data we can reason clinical implications in the prevention of weight gain for the improvement of public health. The potential belief of individuals with an external locus of control that personal actions do not influence weight gain could be counteracted with education. Our findings suggest that among the range of factors influencing such choices, self-esteem may play a key role. Strategies to promote social and emotional aspects of learning, including the promotion of self-esteem, are central to a number of recent policy initiatives (see for example, [47,48]). Our findings suggest that approaches of this kind may carry positive benefits for physical health as well as for other aspects of children's development and that, as is currently proposed, programmes of this kind should be implemented at primary as well as secondary school ages. These would specifically be concerned with reinforcing the message that lifestyle choices can determine BMI. Children with low self-esteem should be encouraged in order to raise it. One important implication of our results is that these actions should be taken before 10 years of age.

## Conclusion

The literature has shown that obesity and low self-esteem are associated through cross-sectional study, and it has been generally thought that low self-esteem is a result of obesity. However, we also concluded that low self-esteem is antecedent to obesity and is a risk factor for weight gain. We also concluded, consistent with the literature concerning eating disorders, that an external locus of control results in disordered and unhealthy eating and lifestyle choices, which in turn leads to weight gain.

## Abbreviations

BMI: body mass index; BCS70: 1970 Birth Cohort Study; SES: socio-economic status.

## Competing interests

The authors declare that they have no competing interests.

## Authors' contributions

AT compiled the dataset from publicly available sources, performed all statistical analyses and drafted the manuscript. DC advised on background knowledge of the manuscript topics and edited the manuscript. BM advised on statistical methods and edited the manuscript. All authors contributed to the study design and coordination through discussion. All authors read and approved the final manuscript.

## Pre-publication history

The pre-publication history for this paper can be accessed here:


